# The prognostic implications and tumor-promoting functions of CHSY3 in gastric cancer

**DOI:** 10.3389/fimmu.2024.1364979

**Published:** 2024-05-15

**Authors:** Han Wang, Junchang Zhang, Zhuoqi Wei, Songyao Chen, Jiabin Zheng, Yong Li

**Affiliations:** ^1^ Guangdong Cardiovascular Institute, Guangdong Provincial People’s Hospital, Guangdong Academy of Medical Sciences, Guangzhou, China; ^2^ Department of Gastrointestinal Surgery, Department of General Surgery, Guangdong Provincial People’s Hospital (Guangdong Academy of Medical Sciences), Southern Medical University, Guangzhou, China; ^3^ Department of Gastrointestinal Surgery, First Affiliated Hospital of Jinan University, Guangzhou, Guangdong, China; ^4^ Digestive Diseases Center, The Seventh Affiliated Hospital of Sun Yat-sen University, Shenzhen, Guangdong, China

**Keywords:** gastric cancer, CHSY3, tumor-associated macrophages, prognosis, tumor immune microenvironment

## Abstract

Chondroitin sulfate synthase 3 (CHSY3) is an important enzyme that regulates glycosylation, but its role in tumors has not been determined. Here, we showed that high CHSY3 expression promotes proliferation in gastric cancer (GC) cells and is associated with poor prognosis in GC patients. We analyzed the immunohistochemistry data of 150 gastric cancer patients to determine the clinicopathological and survival significance of CHSY3. Immunofluorescence was used to detect the colocalization of CHSY3 with infiltrating immune cells. Additionally, CHSY3 was predominantly found in tumor tissues and showed higher abundance compared to matched adjacent tissues. High CHSY3 expression was associated with more advanced tumor stage, higher recurrence risk and worse survival. Immunohistochemistry and bioinformatic analysis revealed that CHSY3 expression was significantly positively correlated with tumor-associated macrophage (TAM) infiltration. Moreover, after knocking down CHSY3, the proliferation of cells was decreased, and the migration ability was reduced, as shown by scratch, monoclonal and transwell assays. In conclusion, this study revealed that CHSY3 has a tumor-promoting effect on GC, suggesting a novel therapeutic strategy against this disease.

## 1 Introduction

Gastric cancer ranks as the fifth most prevalent cancer and the third leading cause of cancer-related mortality worldwide (1). Surgical resection with adjuvant chemotherapy is the main treatment for gastric cancer, but the prognosis remains unfavorable due to chemotherapy insensitivity and the emergence of chemoresistance, frequently resulting in postoperative recurrence (2). Notably, modifications in the tumor immune microenvironment hold predictive value for patient prognosis (3, 4). Hence, accurately forecasting patient responses to therapy emerges as a crucial challenge.

One of the common posttranslational modifications of proteins is glycosylation (5, 6), a process involving the transfer of sugar chains to form glycosidic bonds between proteins and specific amino acid residues, facilitated by glycosyltransferases. These modifications are closely linked to the development of malignant tumors and the prognosis of cancer patients, achieved through the alteration of sugar chains (7). In addition, aberrant alterations in glycosylation on the surface of tumor cells lead to tumor immune evasion, thereby providing new immune checkpoints (ICs) for immunotherapy (8, 9).

In this study, we revealed the function and expression of CHSY3 in gastric cancer. High CHSY3 expression was associated with poor patient prognosis, and the experiments showed that CHSY3 expression regulated the proliferation and migration of gastric cancer cells, and increased the infiltration of tumor-associated macrophages. In conclusion, these data suggest that CHSY3 can promote gastric cancer development and underscore its potential relevance as a prognostic biomarker for gastric cancer treatment.

## 2 Methods

### 2.1 LinkedOmics Database analysis

The LinkedOmics Database is a public portal that includes multiomics data from all 32 TCGA cancer types and 10 Clinical Proteomics Tumor Analysis Consortium (CPTAC) cancer cohorts, this portal provides biologists and clinicians with a unique platform for accessing, analyzing, and comparing multiomics data within and across tumor types. The genes related to CHSY3 were screened from the TCGA stomach adenocarcinoma (STAD) cohort by the LinkFinder module in the database, and the Pearson correlation coefficient was used to test the results; the results are shown as volcano plots and heatmaps. Functional module analysis of Gene Ontology biological process (GO_BP) and Kyoto Encyclopedia of Genes and Genomes (KEGG) pathway enrichment analysis (GSEA) of the LinkInterpreter module.

### 2.2 K−M plotter (gastric cancer)

KM plotter (http://kmplot.com/analysis/) was used to evaluate the survival prognosis associated with related genes by mapping the survival curve using 1,065 GC samples with an average follow-up of 33 months. The prognostic significance of CHSY3 in GC, as indicated by overall survival (OS), first progression (FP), and postprogression survival (PPS), was investigated using this database. The hazard ratio (HR) with 95% confidence intervals (CIs) was also estimated, as was the log-rank p value. p < 0.05 indicated statistical significance.

### 2.3 TIMER database analysis

TIMER (https://cistrome.shinyapps.io/timer/) and TIMER2.0 (https://timer.cistrome.org/) is a web-based interactive platform for the systematic analysis of immune infiltration in various malignancies. We investigated the expression of CHSY3 in various malignancies and the relationship between CHSY3 and TIL expression through gene modules. Furthermore, the link between CHSY3 expression and gene signatures of TILs, including CD8^+^/CD4^+^ T cells, tumor-associated macrophages (TAMs), M1 macrophages, M2 macrophages, T cells, and related subtypes has been analyzed. An expression scatter plot between Spearman’s correlation and estimated statistical significance for a pair of genes for GC was constructed using the correlation module. The levels of gene expression are represented as log2 RSEM.

### 2.4 Immunohistochemistry

This study involved the analysis of 150 paraffin-embedded gastric cancer (GC) specimens obtained from the Shanghai Outdo Biotech Company. The formalin-fixed and paraffin-embedded sections were deparaffinized with xylene and then rehydrated. Antigen retrieval was performed with Tris/EDTA buffer (pH 9.0) for 20 min at 95°C in paraffin-embedded tissue sections. The slides were incubated with antibodies against CD68 (1:200; Cell Signaling Technology, #97778) and CHSY3 (1:100; Novus, NBP1-85626) overnight at 4°C. The following steps were performed in accordance with the protocols provided by the manufacturer of the DAB Kit (DAB-0031, Maxim, China). Multiplex immunofluorescence was performed following the instructions of the PANOVUE kit (10234100050). Images of the tissues were observed and captured using a KFBIO Digital Slide Scanner.

### 2.5 Analysis of the DNA methylation status of the CpG islands of the CHSY3 gene

The DNA methylation status at the CpG sites of the CHSY3 gene was analyzed in the STAD-TCGA datasets using the MethSurv database (https://biit.cs.ut.ee/methsurv/). Furthermore, the prognostic value of the CpG methylation status of CHSY3 was evaluated in GC samples. Moreover, the association between the CpG methylation status of CHSY3 and overall survival (OS) in patients with GC was also evaluated. Moreover, the genetic alterations of CHSY3 from TCGA cancers were explored via the cBioPortal (https://www.cbioportal.org/) and are displayed as alteration frequencies.

### 2.6 Western blot assay

Cells and tissue samples were collected for protein extraction. A BCA protein assay kit (Thermo Scientific™, #23225, USA) was used to evaluate the protein concentration. The proteins were separated via sodium dodecyl sulfate–polyacrylamide gel electrophoresis. The polyvinylidene fluoride membrane containing proteins was blocked with 5% milk. Then, specific primary antibodies (CHSY3, Affinity, 1:1000; GAPDH, Proteintech, 1:10000) were applied to the membrane at 4°C overnight. After the membranes were incubated with secondary antibodies, an enhanced chemiluminescence (ECL) Western blotting Substrate (180-5001, Tanon, China) was used to detect the proteins.

### 2.7 Wound-healing and colony formation assays

AGS cells were cultured in plates for 24 hours. A pipette tip was used to draw the surface of the cell layer. A microscope was used to capture images at 0 h, 12 h and 24 h after injury. The distance to the injury area at 24 h was measured. To perform the colony formation assay, 1000–1500 cells were seeded into six-well plates and cultured for approximately 14 days. Cell colonies were fixed with 4% formaldehyde and stained with 0.1% crystal violet for 10 min.

### 2.8 Transwell assay

The cells were mixed with serum-free media and injected into the upper layer. The outside of the transwell chamber was filled with complete medium. After the cells were cultured under suitable conditions for 48 h, the Transwell chambers were removed for fixation and staining with 4% paraformaldehyde and 0.1% crystal violet (Solarbio, China). The cells on the bottom of the chambers were counted.

### 2.9 Immunofluorescence analyses

According to standard protocols, cells were fixed and incubated with primary antibodies (at a 1:100 dilution), fluorescent dye-conjugated secondary antibodies, and DAPI.

### 2.10 Statistical analysis

SPSS 22.0 (IBM, USA) was used for statistical analyses. The data were analyzed with Student’s t test or one-way analysis. All the results are displayed as the mean ± SD. P <0.05 was regarded as statistically significant.

## 3 Results

### 3.1 The expression of CHSY3 across cancers

**Figure 1:**
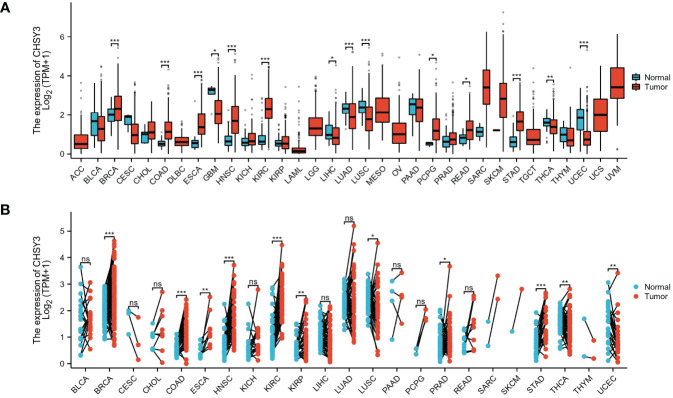
The pancancer mRNA expression of CHSY3. **(A)** The mRNA expression of CHSY3 in 33 tumors in the TCGA database. **(B)** Expression of CHSY3 in paired samples of 23 tumors in the TCGA database. ACC, adrenocortical carcinoma; BLCA, bladder urothelial carcinoma; BRCA, breast invasive carcinoma; CESC, cervical and endocervical cancers; CHOL, cholangiocarcinoma; COAD, colon adenocarcinoma; DLBC, lymphoid neoplasm diffuse large B-cell lymphoma; ESCA, esophageal carcinoma; GBM, glioblastoma multiforme; HNSC, head and neck squamous cell carcinoma; KICH, kidney chromophobe; KIRC, kidney renal clear cell carcinoma; KIRP, kidney renal papillary cell carcinoma; LAML, acute myeloid leukemia; LGG, brain lower grade glioma; LIHC, liver hepatocellular carcinoma; LUAD, lung adenocarcinoma; LUSC, lung squamous cell carcinoma; MESO, mesothelioma; OV, ovarian serous cystadenocarcinoma; PAAD, pancreatic adenocarcinoma; PCPG, pheochromocytoma and paraganglioma; PRAD, prostate adenocarcinoma; READ, rectum adenocarcinoma; SARC, sarcoma; SKCM, skin cutaneous melanoma; STAD, stomach adenocarcinoma; STES, stomach and esophageal carcinoma; TGCT, testicular germ cell tumor; THCA, thyroid carcinoma; THYM, thymoma; UCEC, uterine corpus endometrial carcinoma; UCS, uterine carcinosarcoma; UVM, uveal melanoma. (ns, p > 0.05; *p < 0.05; **p < 0.01; ***p < 0.001).

To ascertain differences in CHSY3 expression between tumor and normal tissues, data from the TCGA database were used. The results showed that CHSY3 was differentially expressed in most tumors, some with high expression and some with low expression (**Figure 1A**). Additionally, the expression of CHSY3 in 23 kinds of tumors with paired samples in the TCGA cohort was also analyzed (**Figure 1B**). In summary, CHSY3 is highly expressed in most cancers.

### 3.2 The expression of CHSY3 in gastric cancer

**Figure 2:**
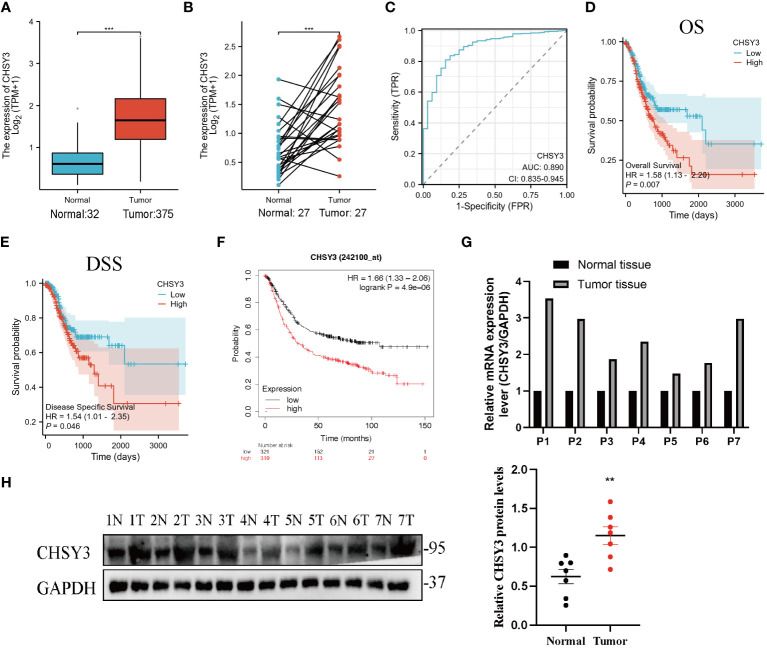
The expression profile of CHSY3 in gastric cancer. **(A)** Increased CHSY3 in gastric cancer tissues compared with normal tissues in the TCGA database. **(B)** Increased CHSY3 expression in gastric cancer tissue compared with matched normal tissue from the TCGA database (n = 27). **(C)** The ROC curve analysis of CHSY3 in GC patients. **(D, E)** High CHSY3 expression was correlated with poor OS and DSS in GC patients according to data from the TCGA database. **(F)** High CHSY3 expression was correlated with poor OS in GC patients according to the Kaplan–Meier plotter database. * P < 0.05, **P < 0.01, ***P < 0.001. **(G)** The mRNA level of CHSY3 in 7 pairs of GC cases and their corresponding adjacent normal tissues. **(H)** Western blotting was performed to detect the protein level of CHSY3 in 7 pairs of GC cases and their corresponding adjacent normal tissues.

As shown in **Figure 2A**, the CHSY3 mRNA level was substantially greater in the GC samples (375 patients) than in the healthy samples (32 patients) (p < 0.05) from the TCGA. As shown in **Figure 2B**, the TCGA database showed that CHSY3 expression was greater in patients than in matched normal tissues (n = 27). Moreover, the area under the curve (AUC) was 0.890 (95% CI=0.835–0.945) for CHSY3 in GC (**Figure 2C**). The above data indicated that CHSY3 expression was strongly increased in GC tissues and may be a potential diagnostic biomarker for GC. Therefore, additional investigations are needed to determine whether CHSY3 expression is associated with tumor outcome. Notably, CHSY3 expression was associated with a favorable outcome in GC patients, and this study showed that increased CHSY3 expression was linked to a worse prognosis in the TCGA GC cohort (OS: HR = 1.58, p = 0.007; DSS: HR=1.54, p=0.046) (**Figures 2D, E**). Moreover, the same results were observed in the 242100-at cohort. High CHSY3 expression correlated with poorer prognosis in GC patients (hazard ratio [HR]=1.66, 95% confidence interval [CI]=1.33 to 2.06, p=0.0000049) (**Figure 2F**). Furthermore, western blotting and qRT-PCR were performed to assess the CHSY3 expression between 7 pairs of GC cases and their corresponding adjacent normal tissues. The results unveiled a notably heightened expression of both CHSY3 mRNA transcripts and proteins in the GC tissues relative to their adjacent healthy tissues (**Figures 2G, H**).

### 3.3 CHSY3 co-expressed genes in gastric cancer

**Figure 3:**
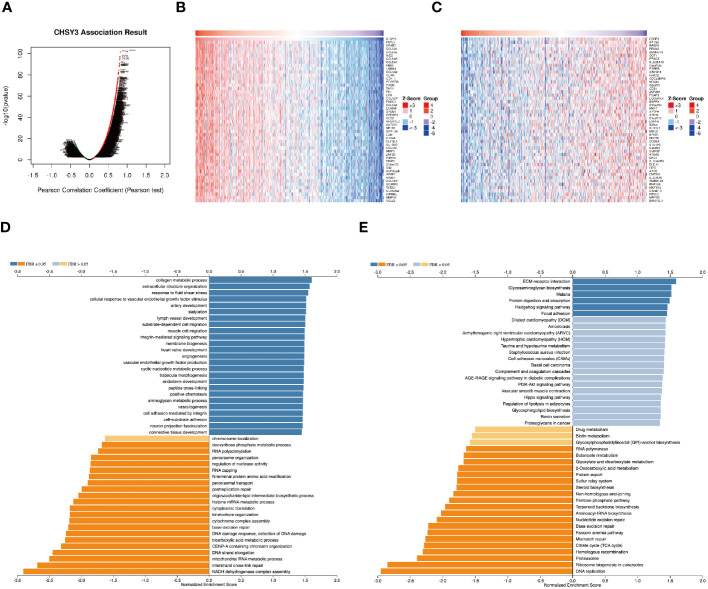
Genes co-expressed with CHSY3 in STAD were analyzed via the LinkedOmics database. **(A)** All genes significantly associated with CHSY3 were identified by Pearson correlation in the STAD cohort. **(B, C)** The top 50 genes positively and negatively related to CHSY3 in STAD are shown by heatmaps. Red represents positively linked genes, and blue represents negatively linked genes. **(D, E)** GO annotations and KEGG pathways associated with CHSY3 in the STAD cohort.

The above results revealed that CHSY3 expression was significantly associated with the prognosis in gastric cancer patients. Next, we explored CHSY3 co-expression networks using the LinkedOmics database to verify the potential function of CHSY3 in tumor tissue (**Figure 3A**). A total of 10689 co-expressed genes were significantly correlated with CHSY3 in GC (FDR < 0.05, P < 0.05, and |cor.| ≥ 0.3). Among the 10689 genes, 6290 were positively correlated with CHSY3 expression, whereas 4399 were negatively correlated with CHSY3 expression. **Figures 3B, C** shows heatmaps of the top 50 genes positively and negatively associated with CHSY3.

GO term annotation revealed that the genes co-expressed with CHSY3 were involved mainly in collagen metabolic processes, extracellular structure organization, response to fluid shear stress, cellular response to vascular endothelial growth factor stimulus, etc. (**Figure 3D**). KEGG pathway analysis revealed enrichment of ECM-rector interaction, glycosaminoglycan biosynthesis, malaria, protein digestion and absorption, and the Hedgehog signaling pathway (**Figure 3E**). These results revealed the wide influence of the CHSY3 expression network on the prognosis of patients with STAD.

### 3.4 Immune infiltration analysis

**Figure 4:**
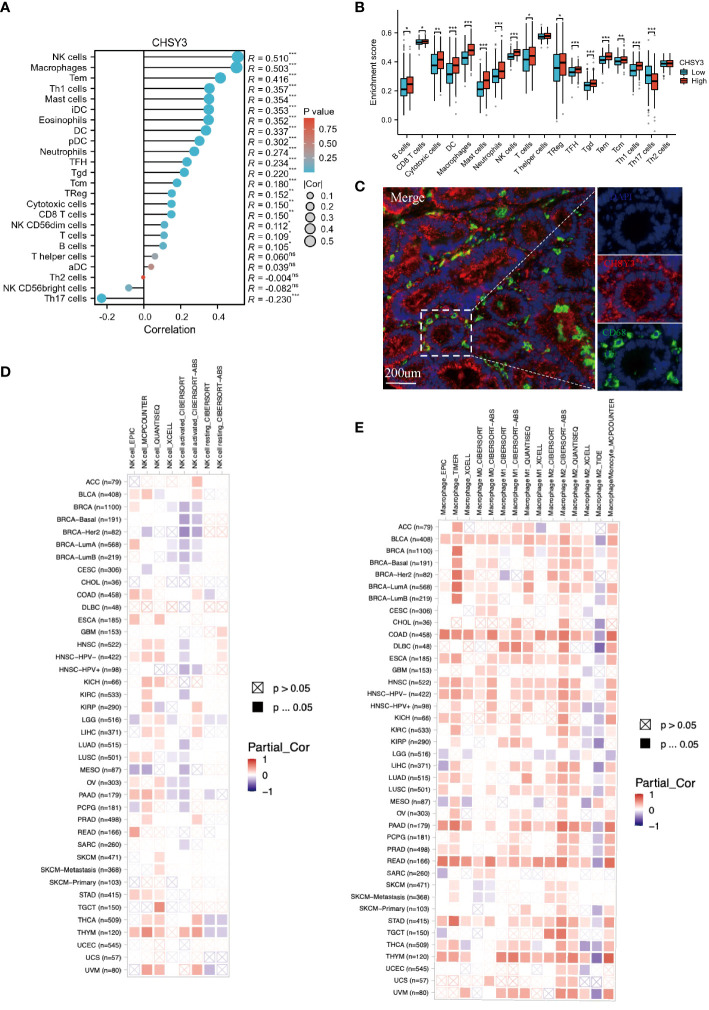
Correlations between CHSY3 expression and immune cell infiltration. **(A)** Relationships between CHSY3 expression and the infiltration levels of NK cells, macrophages, B cells, CD4^+^ T cells, CB8^+^ T cells, neutrophils, and dendritic cells in human cancers. **(B)** Relationship between the high/low expression of CHSY3 and immune cells infiltration. **(C)** mIHC staining demonstrating the colocalization of CHSY3 (red) with macrophages (green). **(D, E)** Heatmaps of the correlations between CHSY3 expression and NK cells and macrophages in the TIMER2 database. *p < 0.05, **p < 0.01, ***p < 0.001.

The immune microenvironment plays a crucial role in the occurrence and development of tumors. The relationship between CHSY3 and the immune microenvironment in GC was studied via the TCGA database, and the results of the R package “GSVA” demonstrated that CHSY3 was positively correlated with different immune cells in these cancers (**Figure 4A**). Furthermore, the relationship between the high/low expression of CHSY3 and immune cell infiltration was analyzed, and the results showed that the up-regulation of CHSY3 expression increased the infiltration level of various immune cells, especially tumor-associated macrophages (**Figure 4B**). By immunofluorescence, we found that CHSY3 (red) and CD68 (a marker of macrophages; green) were correlated, which was consistent with the findings of previous studies (**Figure 4C**). Heatmaps showing the correlations between CHSY3 expression and NK cells (**Figure 4D**) and macrophages (**Figure 4E**) are shown.

### 3.5 Correlation of CHSY3 expression with immune infiltration level and cumulative survival in patients with GC

**Figure 5:**
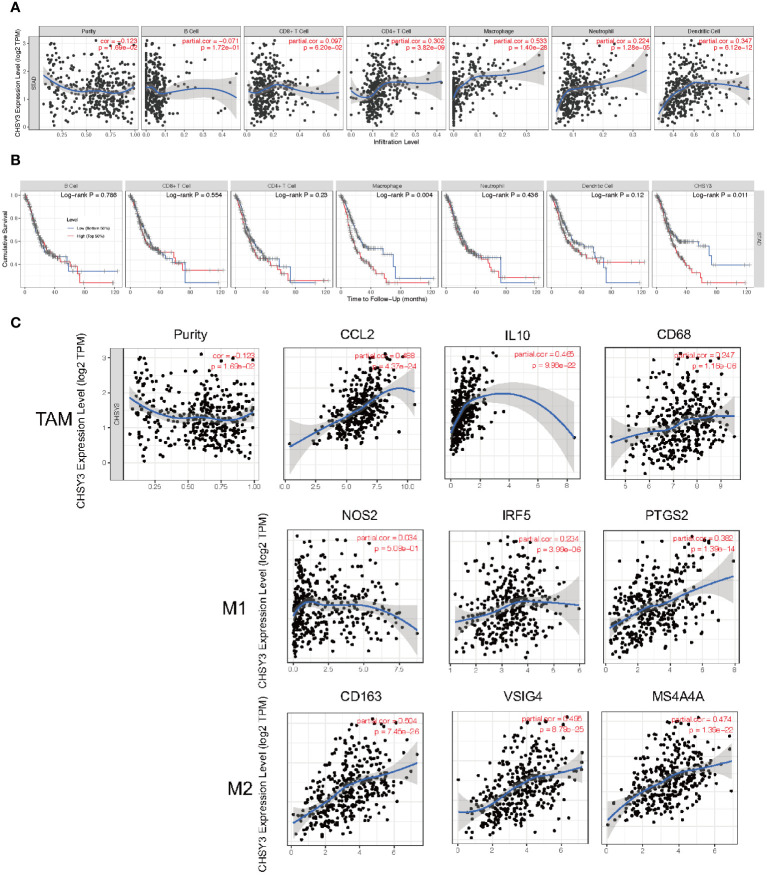
Correlation of CHSY3 expression with immune infiltration level in STAD. **(A)** The CHSY3 expression level was significantly correlated with the infiltration of B cells (r = −0.071, p = 0.172), CD8^+^ T cells (r = 0.097, p = 0.062), CD4^+^ T cells (r =0.302, p = 0.000), macrophages (r = 0.533, p = 0.000), neutrophils (r = 0.224, p = 0.000) and DCs (r = 0.347, p = 0.000) in patients with STAD. **(B)** Cumulative survival was related to B cells (p = 0.786), CD8^+^ T cells (p = 0.554), CD4^+^ T cells (p = 0.23), macrophages (p = 0.004), neutrophils (p = 0.436) and DCs (p = 0.011) in patients with STAD. **(C)** Scatterplots of the correlations between CHSY3 expression and the gene markers of TAMs and M1 and M2 macrophages in STAD.

As mentioned above, several tumor-infiltrating lymphocytes are independent predictors of cancer survival; thus, we investigated the association between CHSY3 expression and immune infiltration levels in GC patients. We selected CHSY3 expression levels that were positively correlated with tumor purity. The results showed that the level of CHSY3 expression was negatively correlated with the infiltration level of B cells (r = −0.071, p = 0.172) and positively correlated with the infiltration level of CD8^+^ T cells (r = 0.097, p = 0.062), CD4^+^ T cells (r = 0.302, p = 0.000), macrophages (r = 0.533, p = 0.000), neutrophils (r = 0.224, p = 0.000) and DCs (r = 0.347, p = 0.000) in GC (**Figure 5A**). Moreover, our findings revealed that B cells (p = 0.786), CD8^+^ T cells (p = 0.554), CD4^+^ T cells (p = 0.23), macrophages (p = 0.004), neutrophils (p = 0.436) and DCs (p = 0.12), but only macrophages, were related to the cumulative survival rate of patients with GC over time (**Figure 5B**). These data strongly indicate that CHSY3 is associated with macrophages infiltration in GC. CHSY3 was significantly associated with the majority of the macrophage marker sets in STAD. Specifically, this study revealed that the TAM markers chemokine ligand (CCL)-2, CD68 and interleukin 10 (IL10) are strongly correlated with CHSY3 in STAD, as are interferon regulatory factor 5 (IRF5) and prostaglandin-endoperoxide synthase 2 (PTGS2) in the M1 phenotype and with CD163, V-Set and immunoglobulin domain containing 4 (VSIG4), and the Membrane Spanning 4-Domains A4A (MS4A4A) in the M2 phenotype (p < 0.001; **Figure 5C**; **Supplementary Table 1**).

### 3.6 CHSY3 is highly expressed in and influences the proliferation and migration of GC cells

**Figure 6:**
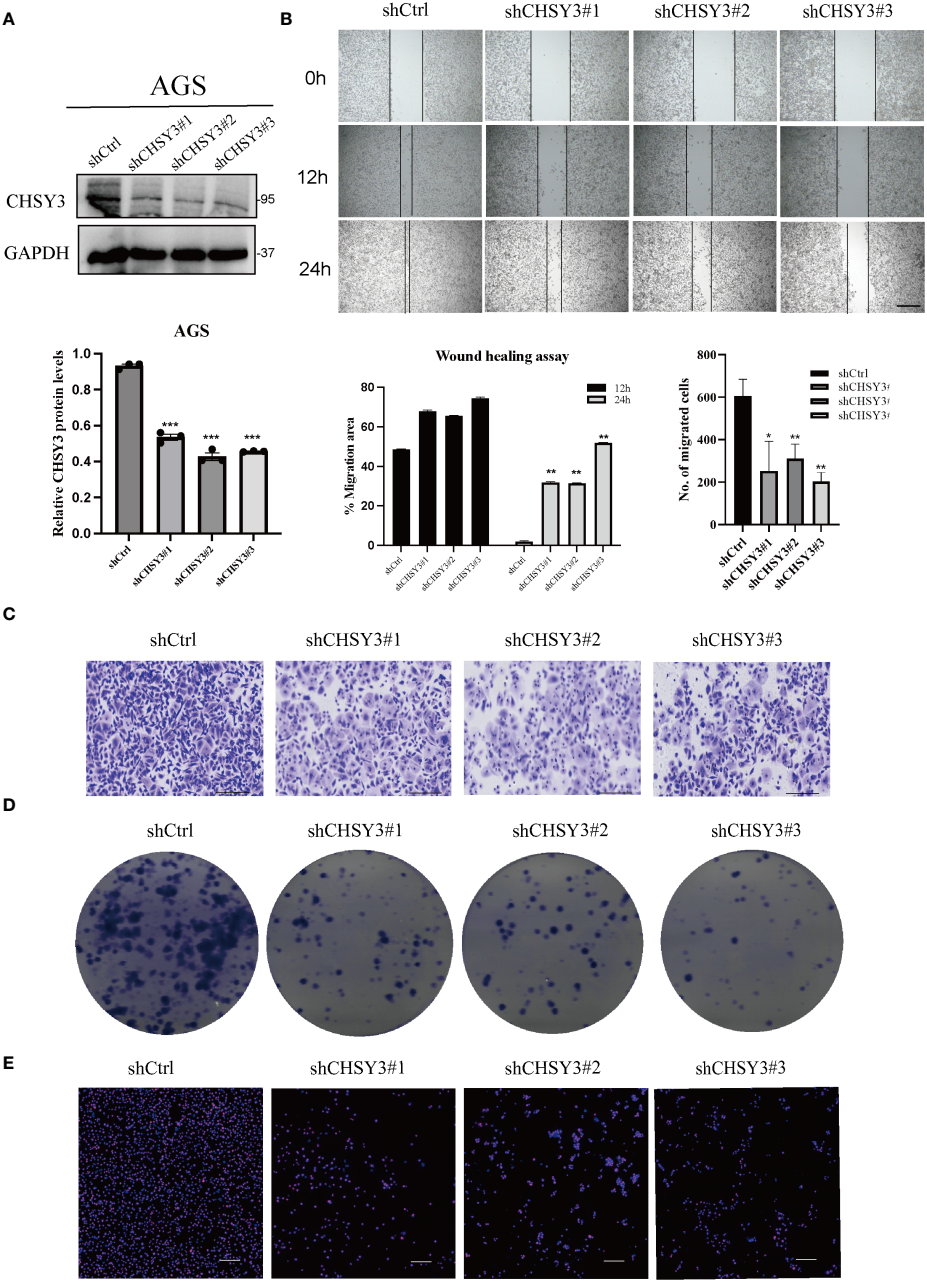
CHSY3 promotes the malignant properties of GC progression **(A)** WB was used to verify the efficiency of the shRNAs. **(B)** Wound healing assays indicated that CHSY3 knockdown could restrain the migration of GC cells. **(C)** Transwell assays showed that CHSY3 knockdown inhibited the migration of GC cells. **(D)** Colony formation assays were used to evaluate the effect of CHSY3 knockdown on the growth of AGS cells. **(E)** IF analysis of the relative expression of Ki-67 in CHSY3-knockdown cells. *p < 0.05, **p < 0.01, ***p < 0.001.

To confirm the function of CHSY3, we designed three shRNAs that targeted different sites of CHSY3. Western blot analysis indicated that the three shRNAs had excellent efficacy in knocking down CHSY3 expression (**Figure 6A**). Moreover, wound healing and Transwell assays revealed that CHSY3 knockdown inhibited the migration of GC cells (**Figures 6B, C**). Colony formation and Ki67 staining assays demonstrated that knockdown of CHSY3 significantly decreased the proliferation of GC cells (**Figures 6D, E**).

**Figure 7:**
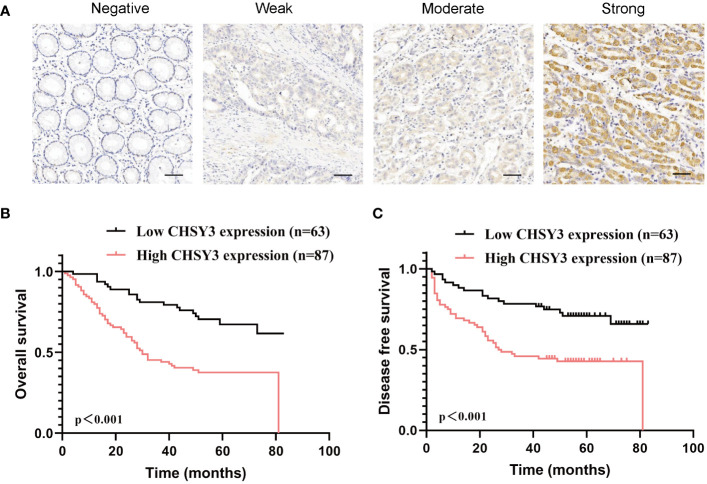
The expression and prognostic value of CHSY3 in gastric cancer **(A)** Gastric cancer tissue was quantified by scoring the staining intensity, which included negative (–) and weak (+) staining and moderate (++) and strong (+ + +) staining. Scale bar = 100 mm. **(B-C)** High CHSY3 expression was correlated with poor OS and DFS in GC patients.

To assess the clinical significance of CHSY3 expression, we examined the protein expression of CHSY3 in GC using immunohistochemistry (IHC) and investigated the relationship between CHSY3 expression and clinicopathological characteristics in GC patients. We found that the expression of CHSY3 was associated with pathological TNM stage, lymph node metastasis, and LVI in gastric cancer patients (**Figure 7A**; **Table 1**). Moreover, we performed survival analysis and showed that increased CHSY3 expression was linked to worse OS and DFS in GC patients (**Figures 7B, C**; **Tables 2**, **3**), which revealed that higher CHSY3 expression was significantly associated with worse prognosis in individuals diagnosed with GC.

**Table 1:** Associations of CHSY3 expression with clinical parameters in GC patients.

Characteristic	CHSY3		
	Low (%)	High (%)	*P*
Age (years)			0.379
>60	28(38.4)	45(61.6)	
≥60	35(45.5)	42(54.5)	
Gender			0.547
Male	40(44.0)	51(56.0)	
Female	23(39.0)	36(61.0)	
Tumor size			0.583
≤5 cm	38(44.2)	48(55.8)	
>5 cm	25(39.7)	38(60.3)	
Borrmann type			0.110
I-II	14(53.8)	12(46.2)	
III-IV	32(36.4)	56(63.6)	
Differentiation			0.191
Well+ moderate	18(52.9)	16(47.1)	
poor	28(40.0)	57(60.0)	
pTNM stage			**0.002**
I-II	34(57.6)	25(42.4)	
III-IV	29(31.9)	62(68.1)	
Depth of invasion			0.098
T1/2	18(54.5)	15(45.5)	
T3/4	45(38.5)	72(61.5)	
Lymph node metastasis			**0.002**
N0	30(60.0)	20(40.0)	
N+	33(33.0)	67(67.0)	
Distant metastasis			0.321
M0	59(43.7)	76(56.3)	
M1	4(26.7)	11(73.3)	
CEA level (μg/L)			0.688
≤5	53(42.7)	71(57.3)	
>5	10(38.5)	16(61.5)	
LVI			**0.035**
Yes	10(27.0)	27(73.0)	
No	47(47.0)	53(53.0)	
PNI			0.330
Yes	5(31.3)	11(68.8)	
No	52(44.1)	66(55.9)	

Bold values indicate P < 0.05.

**Table 2:** Univariate and multivariate analyses for OS in GC patients.

Variable	Univariate			Multivariate		
	HR	95%CI	*P*	HR	95%CI	*P*
Age (years)						
≥60 vs.=60	0.600	0.327-1.101	0.099			
Gender						
Male vs. Female	1.968	1.104-3.509	**0.022**	1.761	1.017-3.049	**0.043**
Tumor size						
>5 cm vs. ≤5 cm	1.719	0.931-3.175	0.084	1.890	1.077-3.314	**0.026**
Borrmann type						
III-IV vs. I-II	3.225	1.066-9.752	**0.038**	3.228	1.073-9.710	**0.013**
Differentiation						
poor vs. Well+ moderate	0.944	0.452-1.972	0.879			
Depth of invasion						
T3-4 vs. T1-2	5.156	0.671-39.594	0.115			
Lymph node metastasis						
N+ vs. N0	3.845	1.554-9.516	**0.002**	3.895	1.648-9.206	**0.002**
CEA level (μg/L)						
>5 vs. ≤5	1.338	0.639-3.014	0.407			
LVI						
Present vs. none	1.057	0.525-2.131	0.876			
PNI						
Present vs. none	1.004	0.360-2.799	0.993			
CHSY3						
High vs. Low	2.493	1.310-4.746	**0.005**	2.272	1.251-4.126	**0.007**

Bold values indicate P < 0.05.

**Table 3:** Univariate and multivariate analyses for DFS in GC patients.

Variable	Univariate			Multivariate		
	HR	95%CI	*P*	HR	95%CI	*P*
Age (years)						
≥60 vs.=60	0.641	0.349-1.177	0.151			
Gender						
Male vs. Female	1.754	0.998-3.084	0.051			
Tumor size						
>5 cm vs. ≤5 cm	1.819	0.984-3.362	0.056	2.159	1.220-3.819	**0.008**
Borrmann type						
III-IV vs. I-II	3.148	1.045-9.484	**0.042**	3.116	1.036-9.370	**0.043**
Differentiation						
poor vs. Well+ moderate	0.954	0.463-1.965	0.899			
Depth of invasion						
T3-4 vs. T1-2	5.249	0.684-40.300	0.111			
Lymph node metastasis						
N+ vs. N0	3.759	1.526-9.258	**0.004**	4.215	1.785-9.953	**0.001**
CEA level (μg/L)						
>5 vs. ≤5	1.213	0.567-2.597	0.618			
LVI						
Present vs. none	1.454	0.737-2.867	0.280			
PNI						
Present vs. none	0.823	0.298-2.273	0.706			
CHSY3						
High vs. Low	2.537	1.346-4.781	**0.004**	2.418	1.333-4.386	**0.004**

Bold values indicate P < 0.05.

### 3.7 DNA methylation analysis of the CHSY3 gene in GC

**Figure 8:**
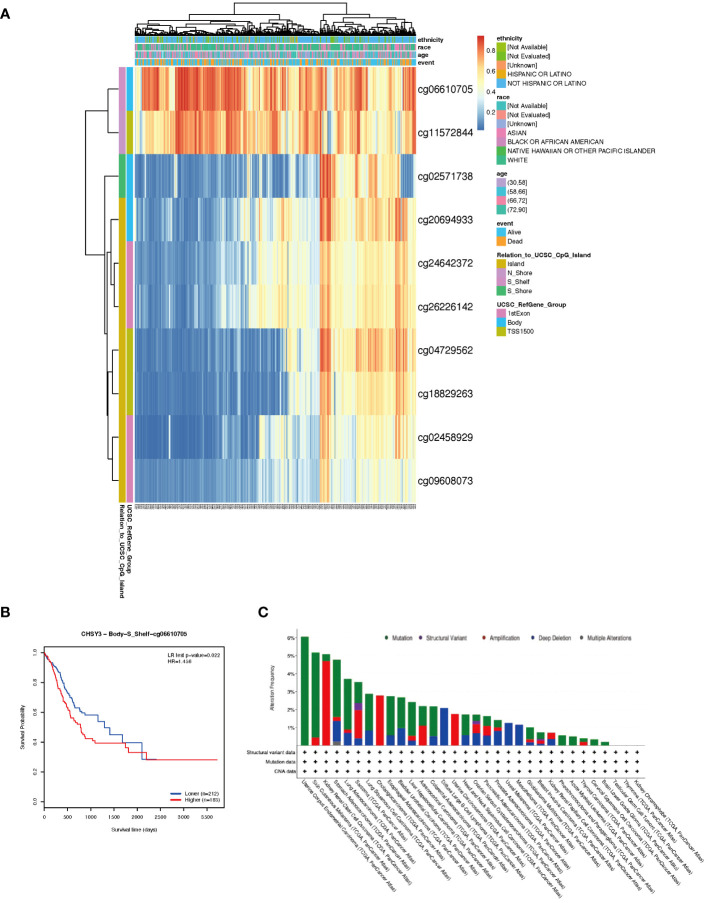
DNA methylation analysis and mutation features of CHSY3 in cancers. **(A)** DNA methylation of CHSY3 in STAD samples from the TCGA. **(B)** Prognostic value of a single CpG in the CHSY3 gene in STAD. The threshold of significance was an LR test p value <0.05. cg06610705 of CHSY3 indicates a significant level of DNA methylation in STAD. **(C)** The alteration frequency and mutation type of CHSY3 (https://www.cbioportal.org/).

DNA methylation is an epigenetic alteration that is related to tumorigenesis and progression (10, 11). DNA methyltransferases that affect CpG island methylation are transcription factors that can suppress or promote cell growth, and the process is reversible (12, 13). A heatmap of DNA methylation clustering of the expression levels of the CHSY3 gene in GC was constructed (**Figure 8A**). Furthermore, the DNA methylation pattern of CHSY3, which has significant prognostic value, was also confirmed, as was that of cg06610705 (**Figure 8B**). Furthermore, the highest alteration frequency of CHSY3 (6%) was observed in uterine corpus endometrial carcinoma patients with “mutation”. In gastric cancer, and most patients had “mutation” or “deep deletion” as the primary alterations (**Figure 8C**).

## 4 Discussion

Immunotherapy stands at the forefront of cancer treatment, demonstrating remarkable success across various cancer types (14). Nonetheless, its efficacy varies among patients (15), potentially due to the intricate immunological microenvironment within tumors (16). Gastric cancer, characterized by a high global mortality rate, typically relies on surgical resection as the primary treatment modality. However, the outcomes of postoperative adjuvant chemotherapy and immunotherapy for advanced gastric cancer remain unsatisfactory. Notably, aberrant glycosylation plays a pivotal role in tumorigenesis and disease progression (1). The CHSY family of glycosyltransferases emerges as a crucial regulator of glycosylation. Specifically, upregulation of CHSY1 has been implicated in promoting the proliferation and metastasis of various cancers including gastric cancer (17), hepatocellular carcinoma (18), glioblastoma (19) and other tumors (20, 21). Recent findings underscore the role of CHSY1 in depleting CD8+ T cells through succinate metabolism activation and the PI3K/AKT/HIF1A pathway, facilitating liver metastasis in intestinal cancer (22). The CHSY2 and CHSY3 genes are identical. High CHSY2 expression has been associated with the occurrence of choriocarcinoma and its metastasis (23). However, only recent reports have shown that CHSY3 expression is associated with the development and metastasis of gastric cancer (24, 25). Like in their study, in which we evaluated the expression of CHSY3 via TIMER, TCGA and other databases, we found that CHSY3 was abnormally highly expressed in a variety of cancers, and compared with paraneoplastic tissues, CHSY3 was more highly expressed in tumors. We found that CHSY3 could promote the progression and migration of gastric cancer cells through cellular experiments, but we found that the expression of CHSY3 was associated with the infiltration of tumor-associated macrophages. We analyzed and verified gastric cancer tissue samples and found that high CHSY3 expression was associated with poorer prognosis and could be an independent risk factor for gastric cancer development.

Chondroitin sulfate, as a core component of the glycosaminoglycan (GAGs) family, plays a crucial role in the occurrence and development of tumors (26). Glycosaminoglycans exert key regulatory functions in the malignant transformation and metastasis of tumors, among which the biosynthesis of chondroitin sulfate is an indispensable part of this process. Specifically, the chondroitin sulfate synthase is essential for the generation of chondroitin sulfate molecules, with chondroitin sulfate synthase-2 being a necessary enzyme to ensure the effective extension of the chondroitin sulfate chain (27, 28). It is noteworthy that chondroitin sulfate has been confirmed as a ligand for the receptor for advanced glycation end products (RAGE) (29), and the role of RAGE in tumor biology is increasingly prominent, particularly in the field of gastric cancer research. The activation status of RAGE is closely associated with its mediation of tumor cell proliferation, enhanced invasive capacity, increased metastatic potential, and poor prognosis in gastric cancer patients (30). These studies further confirm that CHSY3 plays an important role in the development of malignant phenotypes in gastric cancer. Therefore, a thorough exploration of the complex interaction mechanisms between glycosaminoglycans, especially chondroitin sulfate synthase, and the RAGE signaling pathway holds the promise of revealing new anticancer therapeutic targets and strategies, thereby effectively inhibiting the progression of various tumors, including gastric cancer. This not only enriches our understanding of the regulatory mechanisms of the tumor microenvironment but also provides a theoretical basis for the development of targeted anticancer therapies.

In this paper, the role of CHSY3 in gastric cancer was shown to be related to the malignant phenotype of gastric cancer through pathological results and cellular experiments, and we also found that the expression of CHSY3 in gastric cancer was related mainly to the infiltration of macrophages. Tumor-associated macrophages (TAMs) are also special kinds of immune cells (31) that can be divided into M1 and M2 types and play important roles in tumor proliferation, migration, invasion, and tumor immune escape (32–34). M1 macrophages have antitumor functions, including direct cytotoxicity (35) and antibody-dependent cell-mediated cytotoxicity (36) (ADCC), to kill tumor cells. M2 macrophages can promote the occurrence and metastasis of tumor cells (37), inhibit the antitumor immune response mediated by T cells, promote tumor angiogenesis, and lead to tumor progression (38, 39). We found that CHSY3 was closely correlated with M2-type macrophage markers, including CD163, VSIG4, and MS4A4A, but the correlation between CHSY3 and M1-type macrophage markers, such as NOS2 and IRF5, was not strong. This finding suggested that CHSY3 is associated with the polarity of tumor-associated macrophages. Overall, we demonstrated that CHSY3 expression in gastric cancer is associated with immune infiltration. It is very important for tumor patients to choose the appropriate treatment by evaluating their prognosis. In recent years, the infiltration of immune cells in the tumor immune microenvironment has attracted increased attention, and we found that patients with high CHSY3 expression had worse OS.

This study has several limitations. First, we did not study the specific mechanism of the effect of CHSY3 on tumor proliferation and migration, which requires further research. Second, it is not sufficient to use only CD68 as a marker for macrophages. Again, we did not perform *in vivo* experiments to verify the function of CHSY3, and additional in-depth studies are needed.

## 5 Conclusions

Our study revealed a correlation between CHSY3 expression and clinical prognosis, immune infiltrates and DNA methylation. Furthermore, we confirmed that CHSY3 was highly expressed in GC cells and contributed to proliferation and migration. These results could lead to the use of a predictive biomarker and an inclusive understanding of CHSY3 expression in multiple tumor types, especially in GC.

## Data availability statement

The original contributions presented in the study are included in the article/**Supplementary Material**. Further inquiries can be directed to the corresponding authors.

## Ethics statement

The studies involving humans were approved by Shanghai Outdo Biotech Company and Guangdong Provincial People’s Hospital. The studies were conducted in accordance with the local legislation and institutional requirements. The participants provided their written informed consent to participate in this study.

## Author contributions

HW: Conceptualization, Data curation, Formal analysis, Investigation, Methodology, Resources, Software, Supervision, Validation, Visualization, Writing – original draft. JuZ: Conceptualization, Data curation, Formal analysis, Funding acquisition, Investigation, Methodology, Resources, Software, Supervision, Validation, Writing – original draft. ZW: Data curation, Investigation, Methodology, Software, Writing – original draft. SC: Conceptualization, Data curation, Methodology, Software, Writing – original draft. JiZ: Funding acquisition, Project administration, Resources, Supervision, Validation, Visualization, Writing – review & editing. YL: Conceptualization, Funding acquisition, Project administration, Resources, Supervision, Validation, Visualization, Writing – review & editing.
