# Supplementary material for: The prognostic implications and tumor-promoting functions of CHSY3 in gastric cancer
Source: Front Immunol. 2024 May 15;15:1364979. doi: 10.3389/fimmu.2024.1364979 (PMC11133601; doi:10.3389/fimmu.2024.1364979)
Supplement: Supplementary file 1 [file Table_1.docx]

**Supplement table 1**

Correlation analysis between CHSY3 and related genes and markers of macrophage in TIMER2.0.

| Description | Gene markers | STAD | | | |
| --- | --- | --- | --- | --- | --- |
|  |  | None | | Purity | |
|  |  | Cor | P | Cor | P |
| TAM | CCL2 | 0.5 | *** | 0.488 | *** |
|  | CD68 | 0.269 | *** | 0.247 | *** |
|  | IL10 | 0.471 | *** | 0.465 | *** |
| M1 Macrophage | INOS2(NOS2) | 0.039 | 0.433 | 0.034 | 0.509 |
|  | IRF5 | 0.235 | *** | 0.234 | *** |
|  | COX2(PTGS2) | 0.38 | *** | 0.382 | *** |
| M1 Macrophage | CD163 | 0.522 | *** | 0.504 | *** |
|  | VSIG4 | 0.498 | *** | 0.495 | *** |
|  | MS4A4A | 0.482 | *** | 0.474 | *** |

STAD, stomach adenocarcinoma; TAM, tumor-associated macrophage; Cor, R value of Spearman’s correlation; None, correlation without adjustment; Purity, correlation adjusted by purity; CCL2, C-C Motif Chemokine Ligand 2; CD68, CD68 Molecule; IL10, Interleukin 10; INOS, Inducible nitric oxide synthase; IRF5, Interferon Regulatory Factor 5; COX2, Regulation of cyclooxygenase 2; CD163, CD163 Molecule; VSIG4, V-Set And Immunoglobulin Domain Containing 4; MS4A4A, Membrane Spanning 4-Domains A4A.
