# Supplementary material for: The prognostic implications and tumor-promoting functions of CHSY3 in gastric cancer
Source: Front Immunol. 2024 May 15;15:1364979. doi: 10.3389/fimmu.2024.1364979 (PMC11133601; doi:10.3389/fimmu.2024.1364979)
Supplement: Supplementary file 2 [file Table_2.docx]

<https://www.jianguoyun.com/p/DY-1vjsQ3b2kDBjkkq0FIAA>
